# Supplementary material for: Neuronal congruency effects in macaque prefrontal cortex
Source: Nat Commun. 2022 Aug 10;13:4702. doi: 10.1038/s41467-022-32382-1 (PMC9365805; doi:10.1038/s41467-022-32382-1)
Supplement: Supplementary file 1 — Supplementary Information [file 41467_2022_32382_MOESM1_ESM.pdf]

Supplementary Information

Neuronal mechanisms underlying congruency effect in macaque prefrontal cortex

Tao Yao, Wim Vanduffel

Correspondence to: wim@nmr.mgh.harvard.edu; tao.yao@kuleuven.be

Supplement Information consistent of:

Supplemental Figure 1 to 5
Supplemental Table 1 to 3

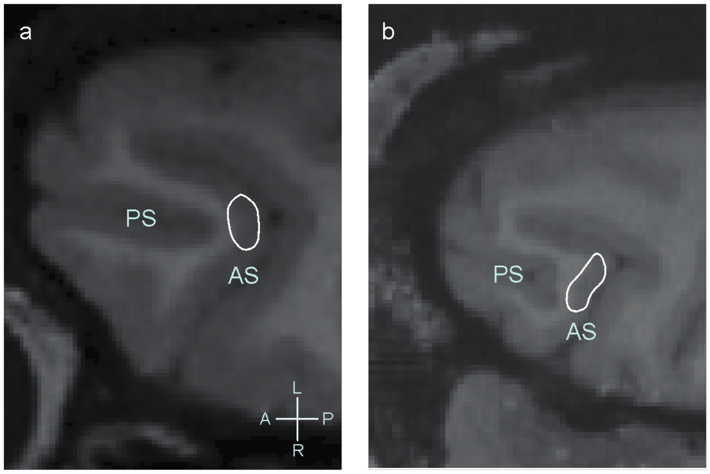

Supplemental Figure 1. Recording areas. All the recording sites in current study were within the areas outlined in white (**a**: Monkey S; **b**: Monkey R). AS: arcuate sulcus, PS: principle sulcus.

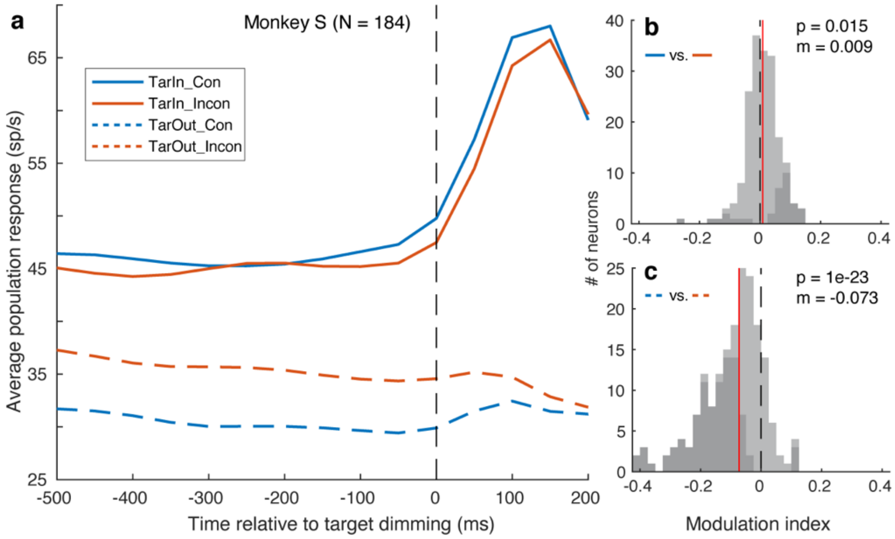

Supplemental Figure 2. The signal and noise change was shown in FEF multi-units (MU) in spatial-rule trials for Monkey S. The MU activity of FEF showed significant both signal and noise change in spatial-rule trials for Monkeys S. Since we did not find a significant signal change for the single-units (SU) in the spatial-rule trials in Monkey S, we performed the same analysis for the multi-units. The MU results showed both a significant signal and noise and change. Please note that the PSTH curves are very similar between SUs and MUs, though the signal change was small. The possible reason why we did not detect a significant signal change at SU level might be due to the small amplitude of the effect relative to the variation across single neurons. The MIs (**b** and **c**) are calculated the same as in Fig. 4 with the average response 500ms prior target dimming in **a**. The p values in **b** and **c** are from the two tailed WSRT. Source data are provided as a Source Data file.

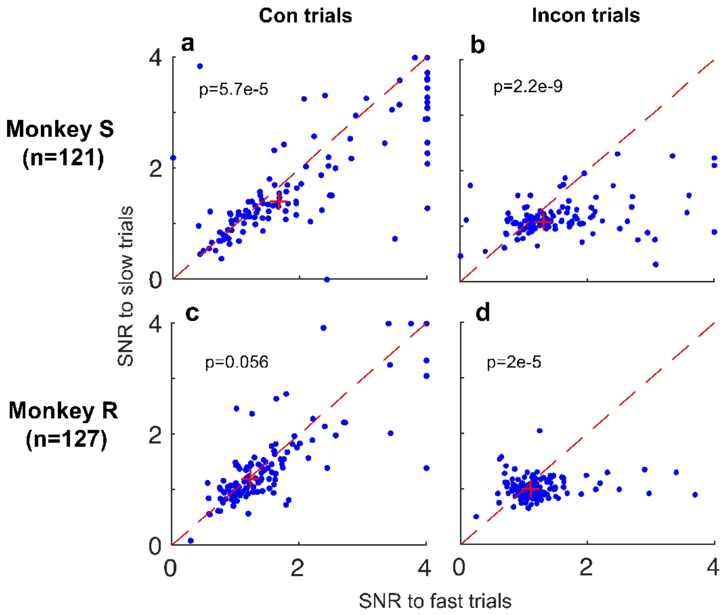

Supplemental Figure 3. The comparison of SNR to slow and fast trials in congruent (a, c) and incongruent (b, d) conditions. The fast trials show higher SNR than slow trials in both congruent and incongruent conditions, suggesting SNR within FEF is sensitive to cognitive modulations indicated by RT. All the p values are from the two tailed WSRT. Source data are provided as a Source Data file.

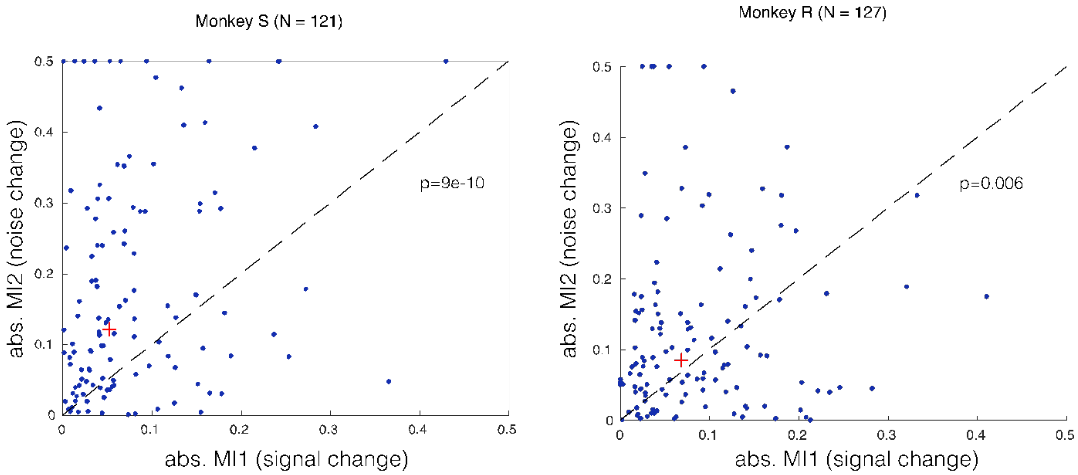

Supplemental Figure 4. Higher absolute modulation index for noise change than signal change in FEF. More neurons showed higher absolute modulation indices when target was out of the RFs (abs. MI2)compared to target was inside the RFs (abs. MI1) suggesting stronger noise change than signal change in FEF. All the absolute MIs higher than 0.5 were plotted as 0.5 in the figure. The red cross in figure indicated the median value of absolute MI1 and MI2. The p values were from Wilcoxon sign rank tests. The calculation of the MI is the same as in Fig 4c-4f. All the p values are from the two tailed WSRT. Source data are provided as a Source Data file.

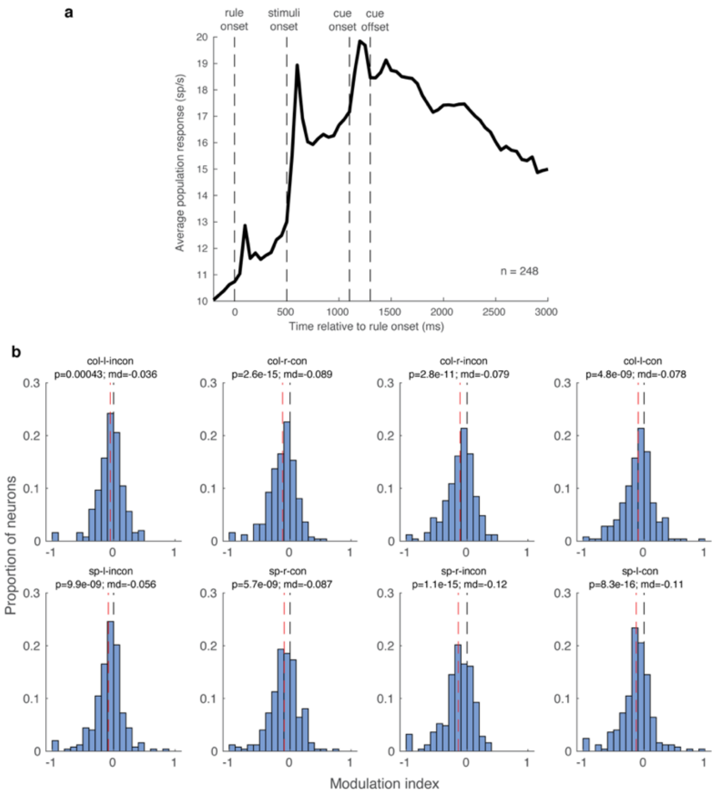

Supplemental Figure 5. ***The neuron response decreases after cue offset along time*.** (**a**) The PSTH of the average neuron response from 248 neurons of two monkeys after the rule cue. The four vertical dashed lines indicates the rule cue onset, stimuli onset, cue onset, and cue offset, respectively. Please note the neuron response decreases after cue offset along time (after the forth vertical line). (**b**) To quantify such decrease, we compared the average neuron response in time window of 300-600ms after cue offset (1600-1900ms after rule cue in **a**) and time window of 1100-1400ms after cue offset (2400-2700ms after rule cue in **a**) by a modulation index (MI: the difference of the two average response divided by the sum of the two, a positive and negative MI indicate increased and decreased response, respectively) for each neuron. We found the distributions of the MIs are significantly shift to the negative side in all conditions (all p<0.0005), indicating the average response in the former time window is significantly higher than the later in all conditions. All the p values are based on the two tailed WSRT. Source data are provided as a Source Data file.

Supplemental Table 1.

		color-cue color	
		red/cyan	pink/blue
**color-cue location**	*left*	congruent	incongruent
	right	incongruent	congruent

The congruent and incongruent conditions in the study. The congruent and incongruent trials are determined by the color and location of the color-cue.

Supplemental Table 2.

	Monkey S				Monkey R			
	**mean ± sem (%)**	**df**	**t**	**p**	**mean ± sem (%)**	**df**	**t**	**p**
sp-l-con	89.6 ± 1.4	11	2.48	0.03	95.6 ± 0.9	16	7.03	$3{\cdot}10^{-6}$
sp-l-incon	84.1 ± 2.4				79.3 ± 1.8			
sp-r-con	97.9 ± 0.6	11	5.89	$1{\cdot}10^{-4}$	96.1 ± 1.1	16	7.12	$2{\cdot}10^{-6}$
sp-r-incon	86.2 ± 1.8				79.2 ± 1.9			
col-l-con	90.2 ± 1.7	11	6.57	$4{\cdot}10^{-5}$	95.8 ± 0.9	16	6.66	$5{\cdot}10^{-6}$
col-l-incon	75.7 ± 2.5				84.6 ± 1.5			
col-r-con	97.6 ± 0.7	11	14.18	$2{\cdot}10^{-8}$	96.1 ± 0.7	16	6.36	$9{\cdot}10^{-6}$
col-r-incon	76.4 ± 1.4				83.4 ± 1.6			

sp: spatial-rule; col: color-rule; l: target at left; r: target at right; con: congruent; incon: incongruent.

The monkey performance accuracy to different conditions in Fig. 3. All the p values are based on the two tailed paired t-test. Source data are provided as a Source Data file.

Supplemental Table 3.

	Monkey S				Monkey R			
	**mean ± sem (ms)**	**df**	**t**	**p**	**mean ± sem (ms)**	**df**	**t**	**p**
sp-l-con	363 ± 6	11	-1.32	0.21	367 ± 7	16	-3.66	$2\cdot10^{-3}$
sp-l-incon	369 ± 8				383 ± 8			
sp-r-con	333 ± 4	11	-0.76	0.46	361 ± 6	16	-6.37	$9\cdot10^{-6}$
sp-r-incon	335 ± 4				382 ± 8			
col-l-con	364 ± 8	11	-1.99	0.07	367 ± 7	16	-0.96	0.35
col-l-incon	371 ± 6				369 ± 7			
col-r-con	335 ± 3	11	-1.49	0.16	363 ± 7	16	-4.31	$5\cdot10^{-4}$
col-r-incon	339 ± 3				374 ± 8			

sp: spatial-rule; col: color-rule; l: target at left; r: target at right; con: congruent; incon: incongruent.

The monkey RTs to different conditions in Fig. 3. All the p values are based on the two tailed paired t-test. Source data are provided as a Source Data file.
