# Supplementary material for: Neuronal congruency effects in macaque prefrontal cortex
Source: Nat Commun. 2022 Aug 10;13:4702. doi: 10.1038/s41467-022-32382-1 (PMC9365805; doi:10.1038/s41467-022-32382-1)
Supplement: Supplementary file 3 — Description of Additional Supplementary Files [file 41467_2022_32382_MOESM3_ESM.pdf]

File name: Supplementary Data 1

Description: Supplementary Data 1 includes the MATLAB code and data and the codes related to all figures in this article. A Readme.txt explaining the code and data is included in the file as well.
