# Supplementary figures and images for: Neuronal congruency effects in macaque prefrontal cortex
Source: Nat Commun. 2022 Aug 10;13:4702. doi: 10.1038/s41467-022-32382-1 (PMC9365805; doi:10.1038/s41467-022-32382-1)

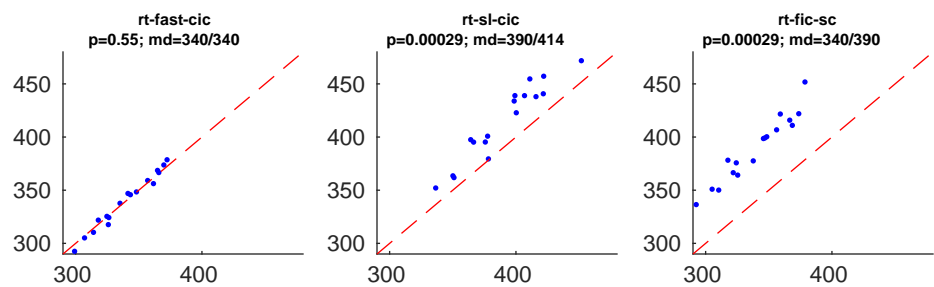

Supplement: Supplementary file 4 — Supplementary Data 1 [file 41467_2022_32382_MOESM4_ESM.zip › data_submit/output/Figure6_Monkey R_RTs.pdf]

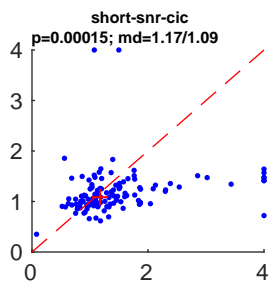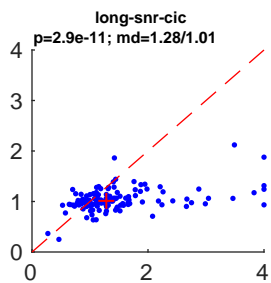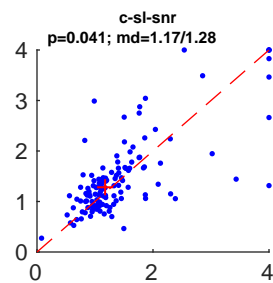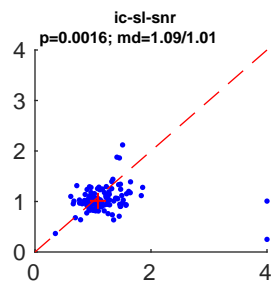

Supplement: Supplementary file 4 — Supplementary Data 1 [file 41467_2022_32382_MOESM4_ESM.zip › data_submit/output/Figure8_Monkey R_snr_con.pdf]

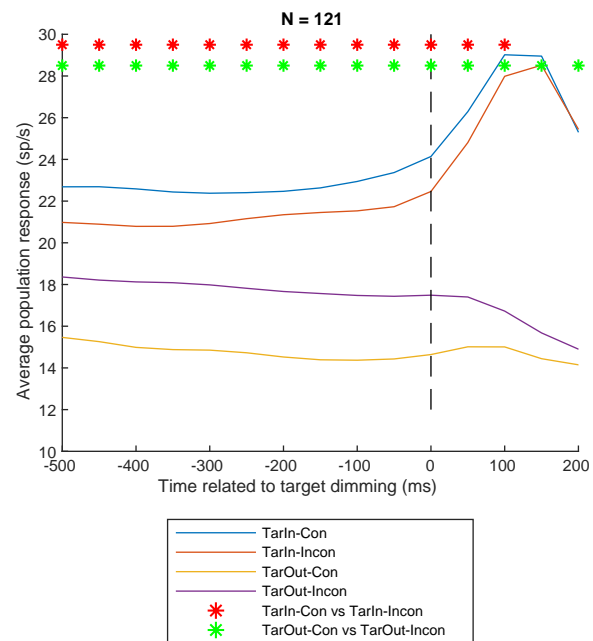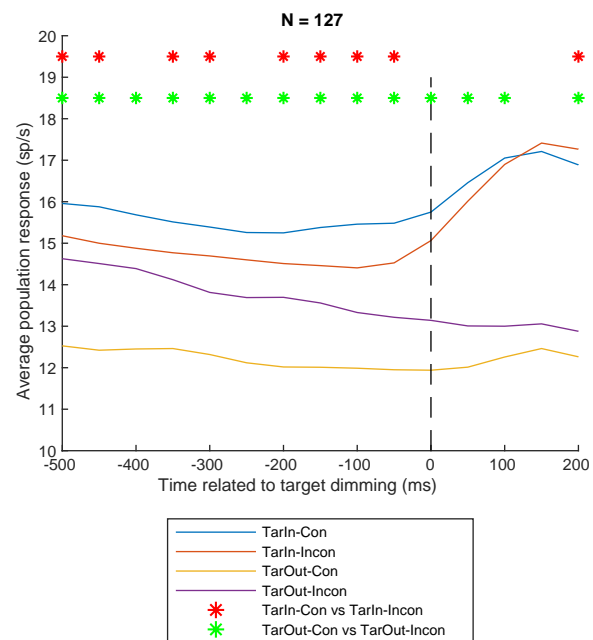

Supplement: Supplementary file 4 — Supplementary Data 1 [file 41467_2022_32382_MOESM4_ESM.zip › data_submit/output/Figure4AB_PSTH.pdf]

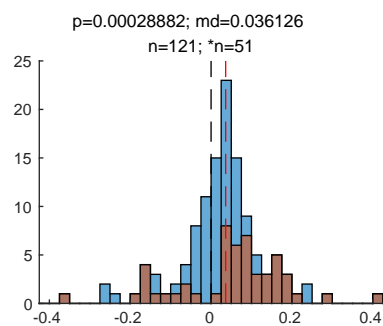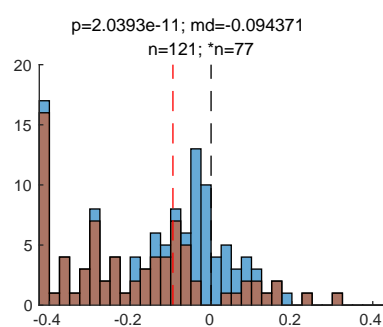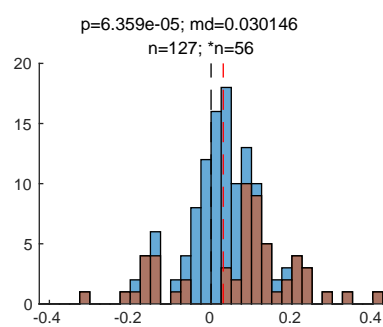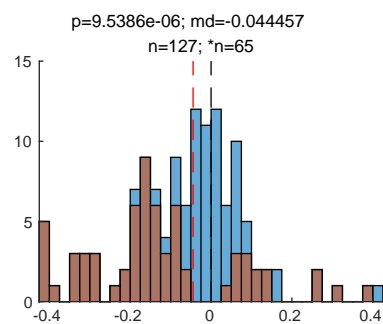

Supplement: Supplementary file 4 — Supplementary Data 1 [file 41467_2022_32382_MOESM4_ESM.zip › data_submit/output/Figure4_hist.pdf]

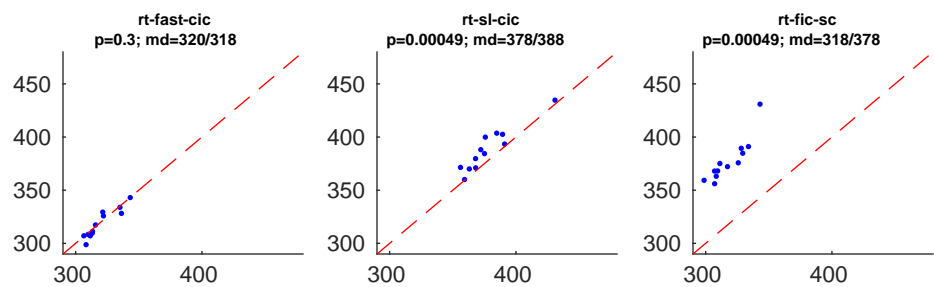

Supplement: Supplementary file 4 — Supplementary Data 1 [file 41467_2022_32382_MOESM4_ESM.zip › data_submit/output/Figure6_Monkey S_RTs.pdf]

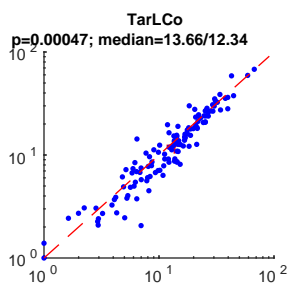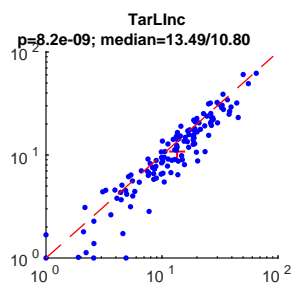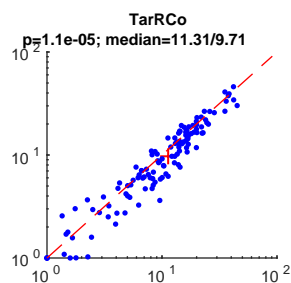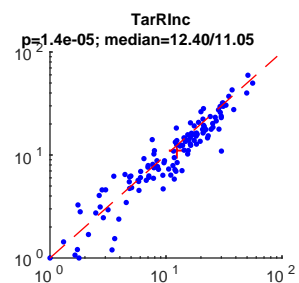

Supplement: Supplementary file 4 — Supplementary Data 1 [file 41467_2022_32382_MOESM4_ESM.zip › data_submit/output/Figure8_Monkey R_FR_sl.pdf]

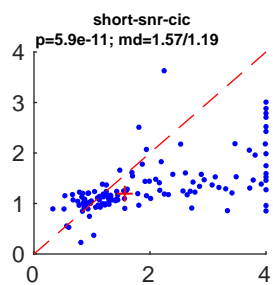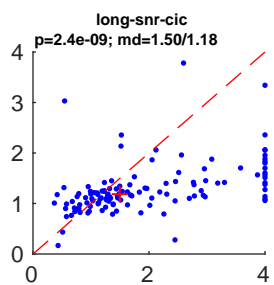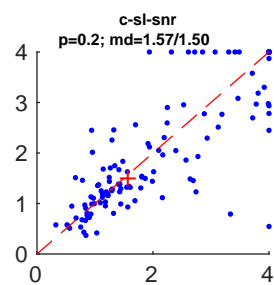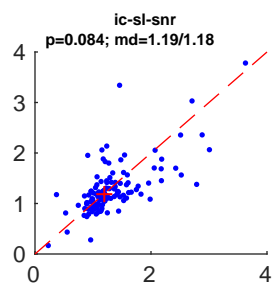

Supplement: Supplementary file 4 — Supplementary Data 1 [file 41467_2022_32382_MOESM4_ESM.zip › data_submit/output/Figure8_Monkey S_snr_con.pdf]

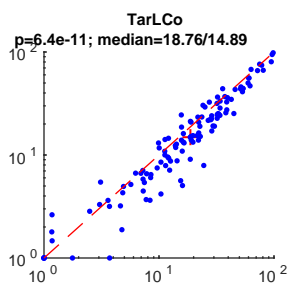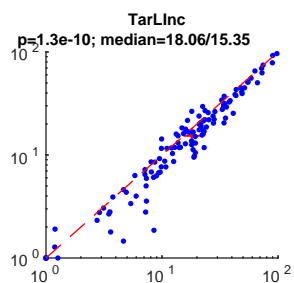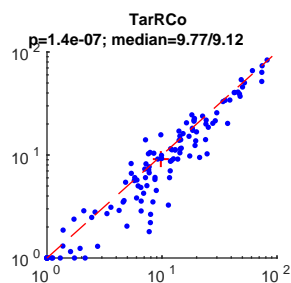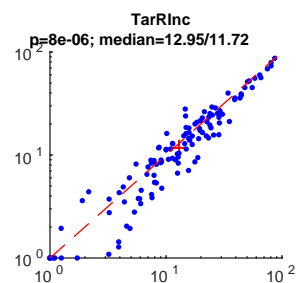

Supplement: Supplementary file 4 — Supplementary Data 1 [file 41467_2022_32382_MOESM4_ESM.zip › data_submit/output/Figure8_Monkey S_FR_sl.pdf]

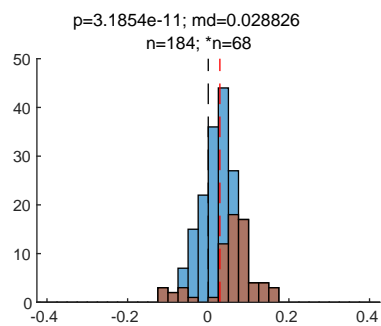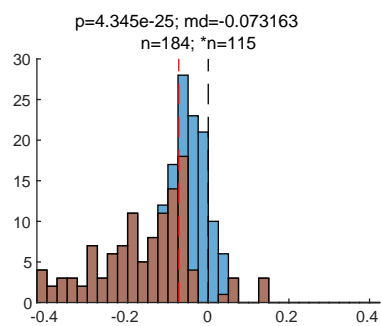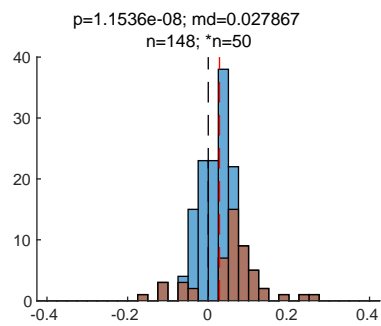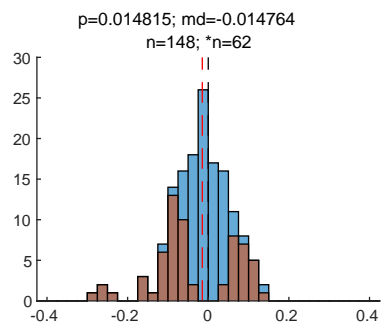

Supplement: Supplementary file 4 — Supplementary Data 1 [file 41467_2022_32382_MOESM4_ESM.zip › data_submit/output/FigureS2_hist.pdf]

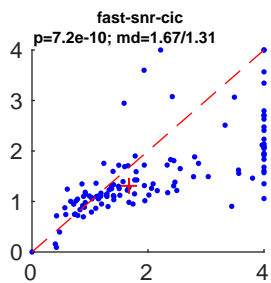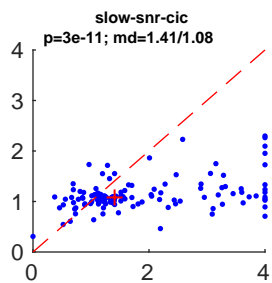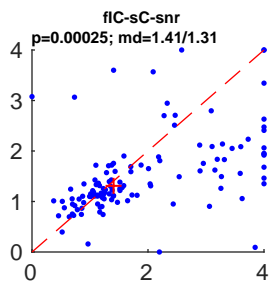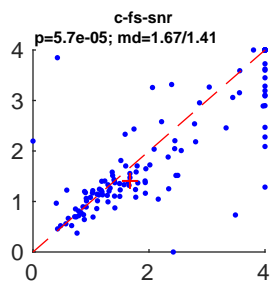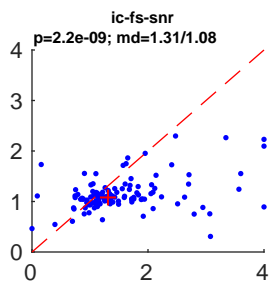

Supplement: Supplementary file 4 — Supplementary Data 1 [file 41467_2022_32382_MOESM4_ESM.zip › data_submit/output/Figure6_Monkey S_SNRs.pdf]

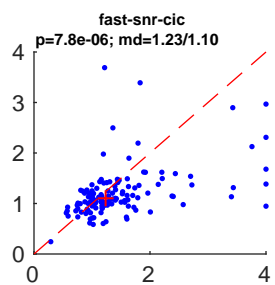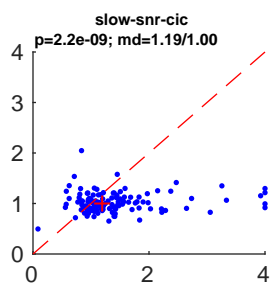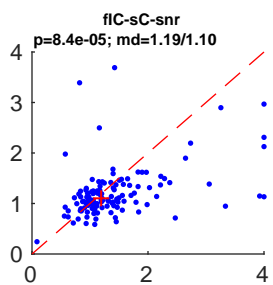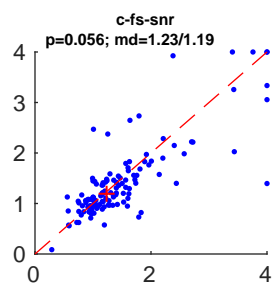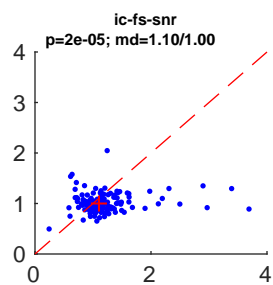

Supplement: Supplementary file 4 — Supplementary Data 1 [file 41467_2022_32382_MOESM4_ESM.zip › data_submit/output/Figure6_Monkey R_SNRs.pdf]

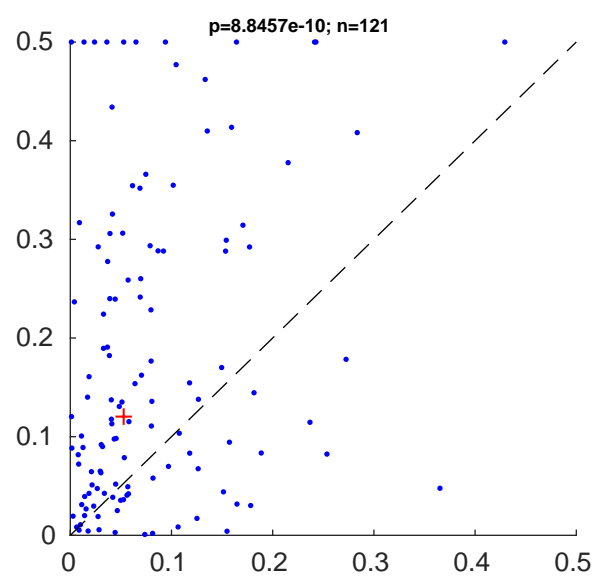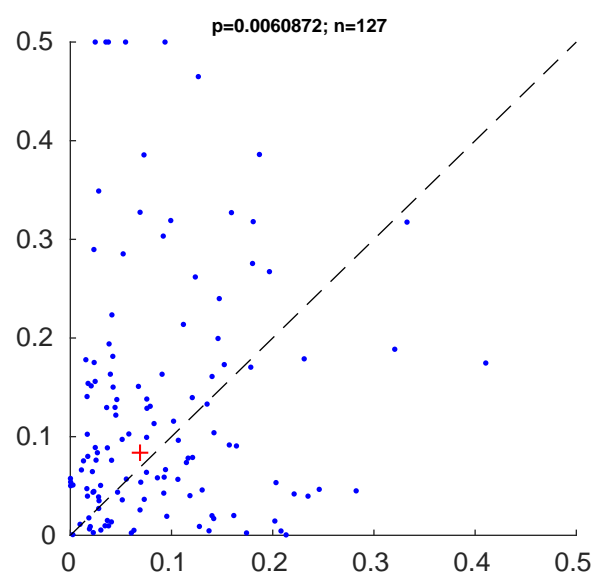

Supplement: Supplementary file 4 — Supplementary Data 1 [file 41467_2022_32382_MOESM4_ESM.zip › data_submit/output/figureS4.pdf]

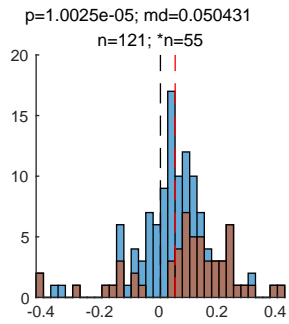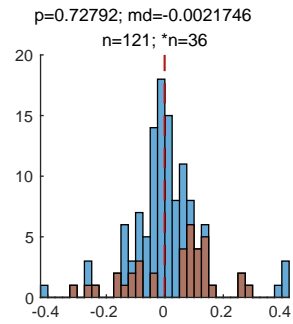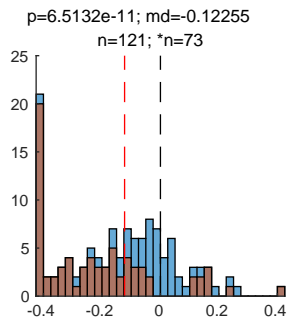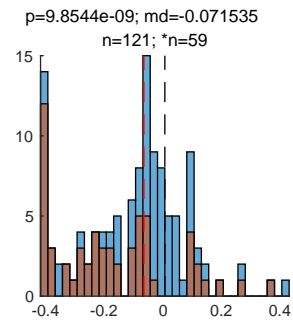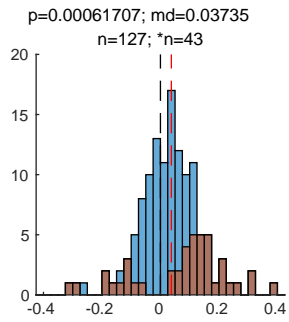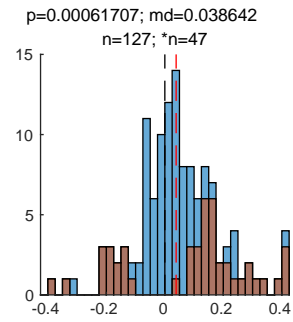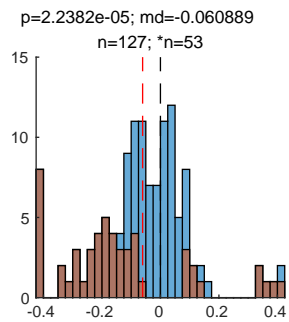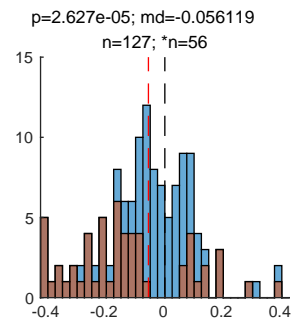

Supplement: Supplementary file 4 — Supplementary Data 1 [file 41467_2022_32382_MOESM4_ESM.zip › data_submit/output/Figure5.pdf]
